# Prolonged hiccups induced by renal infarction: a case report

**DOI:** 10.1186/s13256-024-04347-z

**Published:** 2024-01-28

**Authors:** Akira Kato, Nobuhiro Sato, Yasuo Hirose, Yuji Nomoto, Seiga Ozaki, Saori Yamaga, Masahiro Yabe

**Affiliations:** 1 Department of Emergency and Critical Care Medicine, Niigata City General Hospital, 463-7 Shumoku, Chuo-Ku, Niigata, Niigata 950-1197 Japan; 2 Department of General Care Medicine, Niigata City General Hospital, 463-7 Shumoku, Chuo-Ku, Niigata, Niigata 950-1197 Japan

**Keywords:** Hiccups, Renal infarction, Case report, Atrial fibrillation

## Abstract

**Background:**

Hiccups are common symptoms that last for less than 48 hours. However, we encountered a case of renal infarction in a patient with prolonged hiccup. The relationship between hiccups and renal infarction is important in differentiating patients with prolonged hiccups.

**Case presentation:**

An 87-year-old Japanese man with atrial fibrillation and receiving antithrombotic therapy presented to the emergency department with prolonged hiccups. The patient discontinued antithrombotic therapy for atrial fibrillation due to subcortical bleeding, after which he experienced right back pain. He was diagnosed with right renal infarction based on computed tomography images, and the antithrombotic therapy was continued. The patient’s hiccups ceased, and he was discharged on hospital day 11.

**Conclusion:**

Hiccups can be induced by various clinical conditions. It is hypothesized that the inflammation of the right kidney infarction stimulated the diaphragm and induced prolonged hiccups in this patient; this theory is supported by the computed tomography images. This case report shows that internal organ diseases irritating the diaphragm can cause hiccups, and renal disease should be considered in patients with prolonged hiccups.

## Background

Hiccups are defined as irregular and rapid repetitions of diaphragmatic cramping. Hiccups typically last less than 48 hours, and most cases last only a few minutes. However, some patients experience prolonged hiccups, which last more than 48 hours. Prolonged hiccups interrupt patients’ daily lives [1]. Hiccups are induced by various clinical conditions such as central nervous system diseases, diaphragmatic irritation, and metabolic diseases [2]. Some studies have reported hiccups due to renal diseases, but there are no studies of hiccups due to renal infarction. This report is the first study to present a patient with renal infarction and prolonged hiccups. The onset of acute renal infarction often involves abdominal or flank pain, nausea, vomiting and hematuria; however, some patients may not experience any symptoms. Hiccups due to renal infarction are rare [3].

## Case presentation

An 87-year-old Japanese man with atrial fibrillation and left internal carotid artery stenosis presented to the emergency department with a 2-day history of hiccups.

Approximately 3 weeks prior to his presentation, he was treated for subcortical bleeding in the left occipital lobe due to amyloid angiopathy. During the treatment for the subcortical bleeding, the patient discontinued apixaban for atrial fibrillation and aspirin and experienced acute right back pain that was not treated. The accurate date of the onset of back pain is uncertain. Five days before his hiccup presentation, the patient was discharged from the hospital, and the apixaban and aspirin were resumed; 2 days prior to his presentation, he developed hiccups. Due to the hiccups, he could not sleep and was taken to the emergency room. He had no history of renal diseases and never worried about prolonged hiccups. He drank a glass of beer for over 60 years and had smoked two packs per day of cigarettes but quit about 30 years ago.

**Fig. 1:**
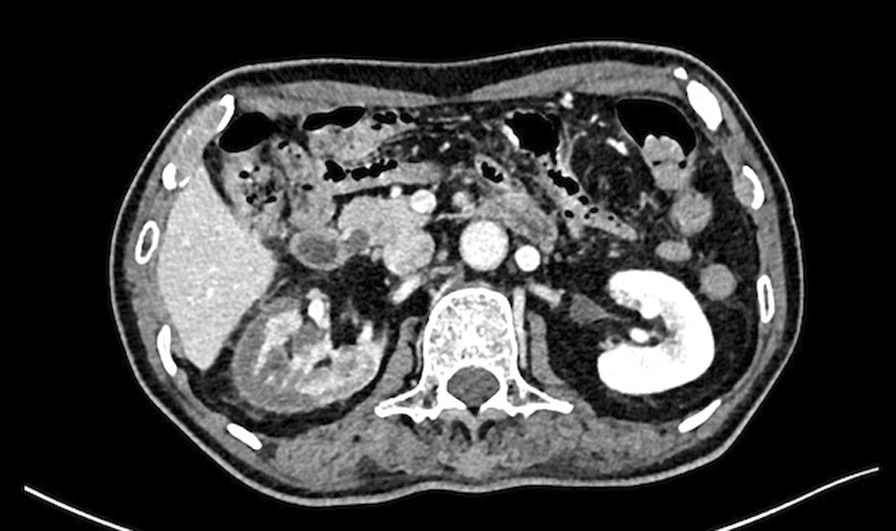
CT revealed multiple poorly-enhanced areas and a cortical rim sign in the right kidney

**Fig. 2:**
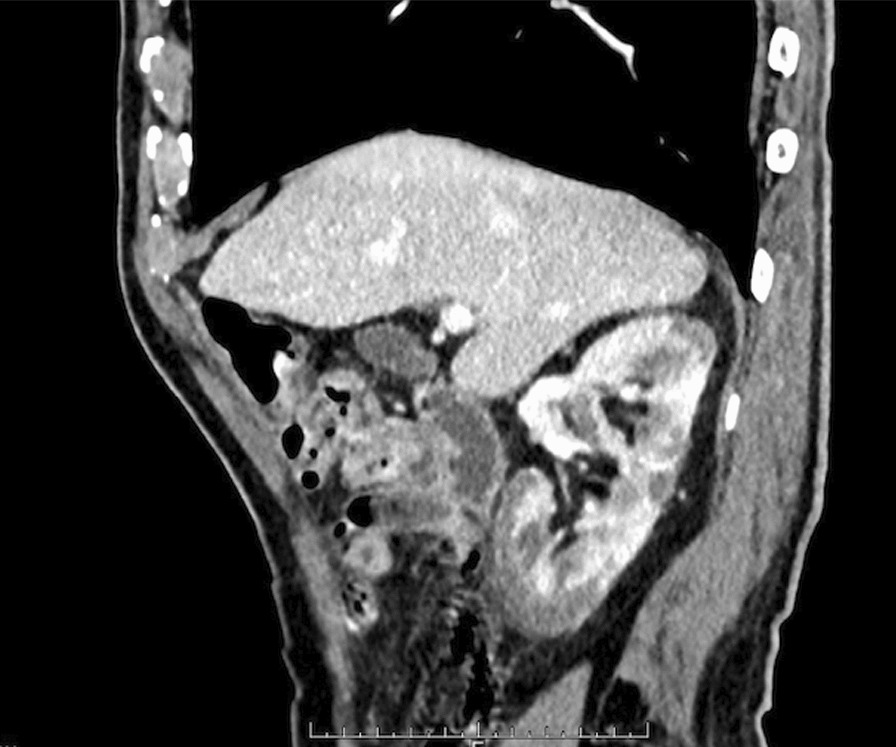
CT revealed that inflammation from the right kidney stimulated the diaphragm

Upon his presentation to the emergency room, his blood pressure was 131/65 mmHg, pulse was 94/minute, respiratory rate was 18 breaths/minute, and oxygen saturation was 98% on room air. No physical abnormalities were noted on examination. His serum lactate dehydrogenase (LDH; 1366 U/l) and C-reactive protein (CRP; 15.53 mg/dl) levels were elevated. Urine tests revealed occult hematuria and proteinuria. On hospital day 1, the patient was referred to the internal medicine department. Although we did a physical examination, he only complained of right costovertebral angle tenderness. Contrast-enhanced computed tomography (CT) revealed multiple poorly enhanced areas and a cortical rim sign in the right kidney (Figs. 1, 2), which suggested renal infarction.

**Fig. 3:**
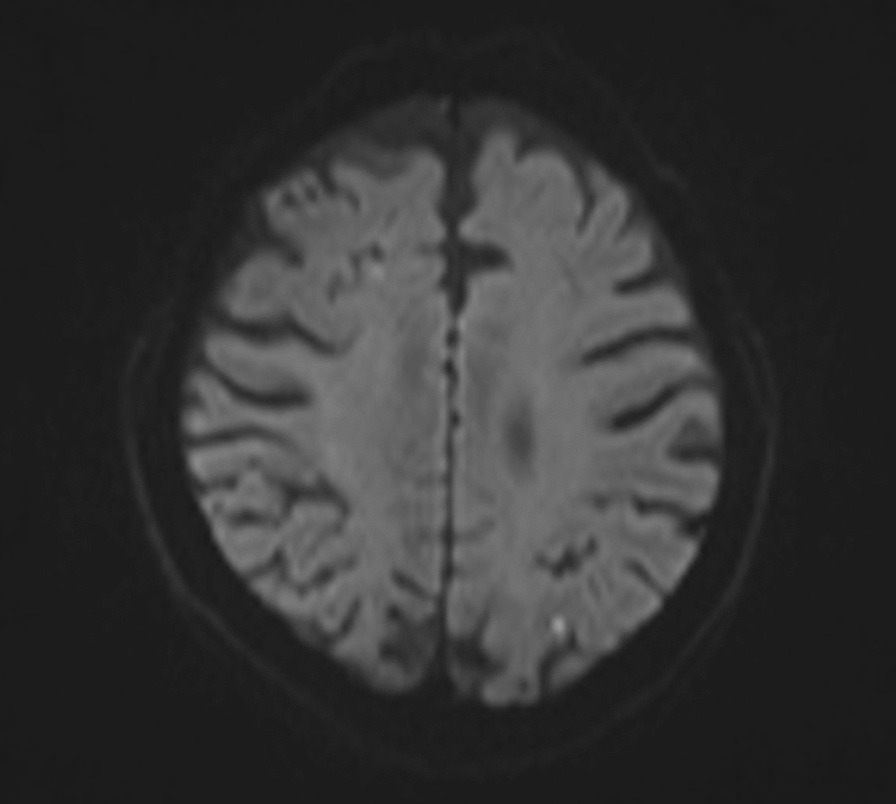
MRI noted only a small old infarction in the left parietal lobe

From the CT findings, which revealed fat stranding from the kidney to the diaphragm, it was hypothesized that inflammation from the right kidney infarction stimulated the diaphragm, inducing prolonged hiccups in reference to previous studies [2]. There were no other findings which could explain the cause of the hiccups. The patient’s typical doses of apixaban (5 mg/day) and aspirin (20.25 mg/day) were continued. Hangeshashinto was administered to as a symptomatic treatment. He was also administered ceftriaxone (2 g every 24 hours) due to the possibility of a urinary tract infection. The patient’s LDH had improved by hospital day 2 (922 U/l), and the patient’s hiccups continued, though their frequency decreased. On hospital day 4, the patient’s LDH (579 U/l) and CRP (5.91 mg/dl) were further improved. His urine and blood cultures were negative, and the ceftriaxone was discontinued. After a gradual decrease in the frequency of the hiccups, they disappeared on hospital day 8. The patient underwent upper gastrointestinal endoscopy and brain magnetic resonance imaging (MRI) to investigate the cause of the hiccups. Ulcerative scarring was noted on endoscopy, and a small, old infarction was noted in the left parietal lobe on MRI (Fig. 3). The patient was discharged on hospital day 11 and continued apixaban (5 mg/day) and aspirin (20.25 mg/day). After discharge from the hospital, he returned to his family physician, and we did not receive notification of any consequences from or recurrence of hiccups.

## Discussion and conclusion

Hiccups are induced by various clinical conditions, including central nervous system diseases, diaphragmatic irritation, irritation of the vagus nerve, medications, and metabolic diseases [2]. These diseases affect the nerve path of the vagus nerve, phrenic nerve, upper spinal cord, brainstem in the medulla oblongata, reticular formation, and hypothalamus in various ways [4]. The vagus nerve consists of thoracic, pharyngeal, and abdominal branches. Intestinal and renal diseases may irritate the abdominal branches of the vagus nerve [2]. Hiccups have been reported in patients with renal abscess [5], giant hydronephrosis [6], acute tubular injury [7], and renal cell carcinoma [8]. Diseases irritating the diaphragm, such as liver abscesses, can cause hiccups [9, 10].

It was hypothesized that the inflammation of the right kidney infarction stimulated the diaphragm and induced prolonged hiccups in this patient, based on the CT images. The cortical rim sign, which indicates zonal enhancement of the renal cortex, is typically detected in patients with renal infarction [11]. This sign represents the outer renal margin, which is perfused by collateral circulation from the gonadal and phrenic arteries [12]. Blood in the urine, elevated serum LDH levels, and elevated serum creatinine levels are important characteristics of renal infarction. In a previous report, 80% of patients with renal infarction had the triad of persistent flank pain, elevated serum LDH levels, and proteinuria [13]. In addition, atrial fibrillation or discontinuation of antithrombotic therapy, which increases the risk of thrombotic events, are associated with renal infarction.

In this patient, subcortical bleeding in the left occipital lobe resulted in the temporary discontinuation of antithrombotic therapy. This patient had a history of atrial fibrillation, which is a high-risk factor for thromboembolism. Right back pain, elevated serum LDH levels, hematuria, and proteinuria are associated with renal infarction. These symptoms and test results are typical for renal infarction [3, 13], but hiccups due to renal infarction have never reported previously. Furthermore, the CT results were consistent with the diagnosis of renal infarction.

Upper gastrointestinal and central nervous system diseases were not detected in this patient, though the CT image revealed fat stranding from the right kidney to the diaphragm. No other findings that can induce hiccups were detected in CT. Discontinuing anticoagulation therapy could not stimulate the nerve path of hiccups, and there was a time lag between discontinuation and the onset of hiccups. These findings were interpreted as inflammation due to an infarction in the right kidney, affecting the diaphragm and inducing hiccups because of the proximity between the kidney and the diaphragm [9]. Moreover, observation of right back pain in another hospital probably contributed to the spread of inflammation. Previous studies have reported hiccups due to renal diseases [5–8], though this is the first report of hiccups due to renal infarction. Therefore, renal disease should be considered in patients with prolonged hiccups. As this is a case report, further research is needed about the neuropathological mechanism between hiccups and diseases of internal organs. Determining the mechanism between renal infarction and hiccups is an interesting topic for further research.

## Data Availability

Data sharing is not applicable to this article as no datasets were generated or analysed during the current study.

## Abbreviations

CT: Computed tomography
LDH: Lactate dehydrogenase
CRP: C-reactive protein
MRI: Magnetic resonance imaging
